# The role of type IV pilus in the interaction of *Neisseria gonorrhoeae* with a corneal epithelium tissue model

**DOI:** 10.1128/iai.00735-25

**Published:** 2026-05-15

**Authors:** Lina Kafuri, Nicola Knetzger, Christian Lotz, Vignesh Parasuraman, Thomas Rudel, Vera Kozjak-Pavlovic

**Affiliations:** 1 Department of Microbiology, Biocenter, Julius-Maximilian University Würzburg, Würzburg, Germany; 2 Translational Center Regenerative Therapies TLC-RT, Fraunhofer Institute for Silicate Research ISC 28474, Würzburg, Germany; 3 Department for Functional Materials in Medicine and Dentistry, University Hospital Würzburg 27207, Würzburg, Germany; University of Virginia, Charlottesville, Virginia, USA

**Keywords:** cornea, *Neisseria gonorrhoeae*, tissue models, type IV pilus, ophthalmia neonatorum

## Abstract

*Neisseria gonorrhoeae,* the causative agent of the sexually transmitted infection gonorrhea, can infect intact corneal epithelium, leading to inflammation, corneal perforation, and blindness. Models for studying this type of gonococcal infection are few and limited. We have tested stratified, polarized cornea models based on an immortalized hTCEpi corneal epithelium cell line for infection research using four derivatives of the *N. gonorrhoeae* MS11 strain that differ in pilus expression. We show that bacterial adherence depends on the functional type IV pilus in the early stages of infection, and that the formation of bacterial microcolonies and biofilms on the model surface leads to tissue destruction. The infection induces a specific cytokine response characterized by an increase in the secretion of IL-8 and tumor necrosis factor (TNF)-α, but not of IL-6. The testing of trifluoperazine, a drug that induces pilus retraction, on infected corneal tissue models showed that the drug strongly diminished the number of adherent gonococci only when applied simultaneously with bacteria, and not when bacteria were allowed to form microcolonies on the tissue surface. Our work describes, for the first time, hTCEpi-based corneal epithelium tissue models as a useful tool for investigating *N. gonorrhoeae* infection, with potential for application in high-throughput studies and drug screening.

## INTRODUCTION

*Neisseria gonorrhoeae* is a Gram-negative diplococcus that causes the sexually transmitted infection gonorrhea. The infection in women is usually asymptomatic, and the risk of transmission to a newborn during birth is considerably high for undiagnosed pregnant women, which is often the case in countries where screening for *N. gonorrhoeae* is lacking (1). Gonococcus (GC) can adhere and colonize the intact corneal epithelium, and is the main cause of ophthalmia neonatorum, an aggressive eye infection characterized by inflammation of the eyelids and the presence of a yellow discharge, which can lead to corneal ulceration, perforation, and blindness (2). Adults are also susceptible to gonococcal eye infections. In recent years, the number of reported cases of eye infections caused by multi-antibiotic-resistant GC has increased due to misdiagnosis of the causative pathogen, delaying proper treatment and leading to permanent corneal injuries (1).

*N. gonorrhoeae* uses different virulence factors to adhere to epithelial cells, including the opacity-associated proteins (Opa) and the type IV pilus. This complex protein machinery consists of many subunits, which together make dynamic filaments with the possibility of retraction and extrusion (3). The main subunit, Pilin or PilE, has a high intra-strain structural variation dependent on the homologous recombination of the large family of *pilS* silent genes, mediated by the Recombinase A (RecA) (4). This high variation not only allows *N. gonorrhoeae* to escape the immune system but also affects how they adhere to epithelial cells. However, PilE is not the only determining factor of the adherence behavior of GC (5). PilC, encoded by two different genes, *pilC1* and *pilC2*, is located at the tip of the pilus where it acts as an adhesin, thus allowing the binding of the bacterium not only to the target tissue (6) but also to other bacteria, which facilitates the formation of microcolonies.

*N. gonorrhoeae* infection research, especially in the genitourinary tract, has been carried out using various models, including humanized hormone-treated mice, *in vitro* models using 2D cell monolayers, and 3D tissue models and organoids. This has allowed for a better understanding of the key factors in gonococcal adherence and invasion (7). Previous infection studies focusing on corneal tissue mostly used human corneal explants (8) or the culture of human corneal epithelial cells on a bovine cornea-derived scaffold (1). Of the different human corneal tissue models established so far, most have been made as an alternative to the Draize irritation test (9), but not specifically for infection studies. The accessibility and high variability of patient-derived primary human corneal epithelial cells can make these models difficult to produce and standardize. As an alternative, different human corneal epithelial cell lines can be used, such as a telomerase-immortalized human corneal epithelial cell line (hTCEpi), which can form stratified epithelium expressing corneal differentiation markers like ZO-1 and cytokeratin 3 when cultured under air-lift conditions (10). Although corneal epithelial cell polarity is relatively understudied, stratified epithelia, such as those found in the cornea, as well as stratified corneal models, generally count as apical-basal polarized (11).

In this work, we used an hTCEpi-based corneal model to study GC infection. We assessed the role of the type IV pilus, using derivatives of the MS11 strain with differences in pilus expression and adherence: MS11 F3 Pil− (Pil^−^, Opa^+^, PilC^+^, RecA^+^), MS11 F3 Pil+ (Pil^+^, Opa^+^, PilC^+^, RecA^+^), MS11 N159 (Pil^+^, Opa^+^, PilC^+^, RecA^−^), and MS11 N191 (Pil^+^, Opa^+^, PilC^−^, RecA^+^). We analyzed the effect of GC infection on tissue permeability, cytotoxicity, and the production of key cytokines, as well as demonstrated the importance of microcolony formation for the damage of the corneal tissue, which is a symptom often observed during GC infection in patients. Lastly, we tested the effect of trifluoperazine, a drug that can induce pilus retraction in *Neisseria meningitidis*. We assessed the pilus retraction effect of the drug in terms of bacterial adherence and IL-8 secretion. We also analyzed the bactericidal activity of trifluoperazine and the induction of GC microcolony disaggregation. In sum, the cornea model used in this study is a suitable and reproducible tool not only for studying basic aspects of the GC eye infection but also for the testing of antimicrobials.

## MATERIALS AND METHODS

### Generation of corneal epithelium tissue models

Human corneal epithelium tissue models were derived from telomerase-immortalized human corneal epithelial cell line (hTCEpi) (Evercyte, Vienna, Austria, #CHT-045-0237) (10). These cells were maintained in E1 medium (EpiLife medium) (Thermo Fisher Scientific, Massachusetts, USA, #MEPI500CA) supplemented with 1% human keratinocyte growth supplement (HKGS) (Thermo Fisher Scientific, Massachusetts, USA, #S0015) and 1% penicillin/streptomycin (P/S) (Sigma-Aldrich, Darmstadt, Germany, #P4333). Cells were seeded into culture flasks at a density of 4–6 cells/cm² and grown to 80%–90% confluence under standard conditions (37°C, 5% CO₂, 95% humidity). The medium was exchanged every 2–3 days.

For passaging, cells were detached with trypsin and centrifuged at 200 × *g* for 5 min. The pellet was resuspended in E2 medium (EpiLife medium supplemented with 1% HKGS, 1% P/S, and 0.48% 300 mM CaCl₂).

Human corneal epithelium models were cultured in BRAND cell culture inserts (BRAND, Wertheim, Germany, #782700) with polycarbonate membranes (0.4 µm pore size and 0.59 cm² growth area). For seeding, 3 × 10⁵ cells in 300 µL E2 medium were added to the apical compartment of each insert and incubated for 2 h under standard conditions (37°C, 95% humidity, 5% CO₂). Subsequently, 1.4 mL E2 medium was added to the basolateral compartment. After 24 h of submerged culture, the medium on the apical side of the inserts was removed, while the medium on the basolateral side was replaced with 1.4 mL E3 medium (EpiLife medium supplemented with 1% HKGS, 1% penicillin/streptomycin, 0.48% CaCl₂, 73 µg/mL ascorbyl-2-phosphate, and 10 ng/mL KGF) to initiate air–liquid interface culture. The medium was exchanged every 2–3 days thereafter.

### Histology

Corneal epithelium models were fixed for 2 h with 4% paraformaldehyde (Morphisto, Offenbach am Main, Germany, #11762.01000) and, after washing three times with 1× phosphate-buffered saline (PBS) (Gibco, Thermo Fisher Scientific, Massachusetts, USA, #10010023), they were disassembled using an 8 mm biopsy puncher. After paraffin embedding, the samples were sectioned into 7 µm-thick slides. After a deparaffinization process, including xylene and rehydration, with a gradient of different concentrated solutions of ethanol, hematoxylin, and eosin staining was done according to standard protocols (12). The stained tissue slides were analyzed with a light microscope (Leica Microsystems, Wetzlar, Germany).

### *Neisseria gonorrhoeae* strains

*N. gonorrhoeae* strains used in this study are MS11 derivatives, and their whole genomes were sequenced using Oxford Nanopore (Microsynth, Balgach, Switzerland). The sequences described in this publication are available at NCBI’s BioProject database under accession number PRJNA1356842.

*N. gonorrhoeae* F3 Pil− (Pil^−^, PilC^+^, Opa^+^, RecA^+^), F3 Pil+ (Pil^+^, PilC^+^, Opa^+^, RecA^+^), N159 (Pil^+^, PilC^+^, Opa^+^, RecA^−^), N191 (Pil^+^, PilC2, Opa^+^, RecA^+^), N931 (Pil^−^, Opa^50^), and N313 (Pil^−^, Opa^57^) were grown for 24 h at 37°C and 5% CO_2_ on plates with Oxoid GC agar base (Thermo Fisher Scientific, Massachusetts, USA, #CM0367) supplemented with 1% (vol/vol) vitamin mix. The day after, approximately 10 single colonies were selected, re-streaked onto a new plate, and grown for 16 h before being used for infection or western blot.

### Infection of corneal epithelium models

Human corneal epithelium models were infected with different *N. gonorrhoeae* strains on day 7 of the development of the models. The bacteria from the plate were taken with a sterile cotton swab and resuspended in 1 mL of DMEM F12 medium (Gibco, Thermo Fisher Scientific, Massachusetts, USA, #31331093). The OD_550_ was measured to calculate the bacterial number, and, taking into account the number of epithelial cells, the models were infected from the apical side for 2 h at an MOI of 20 in DMEM F12 medium. Infection medium was collected from the models and centrifuged to collect the unattached bacteria (2 h bacterial samples). The models were then washed three times with 1× PBS and incubated under airlift conditions until further evaluation at different time points (2, 24, and 72 h). To collect the bacteria after 72 h of infection, infected models were solubilized using 1% (wt/vol) saponin solution (Sigma Aldrich, Darmstadt, Germany, #47036) for 30 min, and the suspension was plated on GC agar plates for cultivation.

### Immunoblotting

Protein content was analyzed by sodium dodecyl sulfate polyacrylamide gel electrophoresis (SDS-PAGE) and western blot. Briefly, the samples were dissolved in Laemmli buffer (62.5 mM Tris, pH 6.8, 2% SDS, 10% glycerol, 5% β-mercaptoethanol, and 0.002% Bromophenol Blue) and separated on a 4%–12% NuPAGE Bis-Tris Precast gel (Thermo Fisher Scientific, Massachusetts, USA, #NP0322BOX). Separated proteins were transferred onto a polyvinylidene fluoride membrane, which was blocked for 1 h with 5% skimmed milk in Tris-buffered saline (20 mM Tris/HCl, pH 7.5 [Carl Roth, Karlsruhe, Germany, #4855.2], 150 mM NaCl [Carl Roth, Karlsruhe, Germany, #0601.2]), followed by the overnight incubation with the pilin SM1 mouse monoclonal antibody (a kind gift from Magdalene Yh So, University of Arizona), Omp85 (custom-made in rabbits against the full-length protein; Davids Biotechnology, Regensburg, Germany), or Opa (in-house made in rabbits) at 4°C. For detection, the membrane was incubated with an HRP-coupled secondary antibody (Biozol, Eching, Germany), and the images were processed with FIJI (13).

### Lipooligosaccharide isolation and analysis

Bacteria grown on a GC agar plate were harvested, washed with PBS, and resuspended in 1 mL PBS, followed by measurement of OD_550_. Bacteria were centrifuged and resuspended in buffer A (0.06 M Tris Base [Sigma-Aldrich, Darmstadt, Germany, #T1503], 1 mM disodium EDTA [Carl Roth, Karlsruhe, Germany, #8043.2], 2% [wt/vol] SDS [Carl Roth, Karlsruhe, Germany, #CN30.7], pH 6.8) to obtain the suspension with 10^10^ bacteria/mL. The suspension was placed in a sonicating bath for 10 min and then incubated at 65°C for 15 min. Following this, Proteinase K (Carl Roth, Karlsruhe, Germany, #7528.1) was added to each tube to a final concentration of 100 µg/mL. The proteins in the bacterial suspension were digested overnight at 37°C, after which the samples were precipitated by adding 1/10 volume of 3 M sodium acetate (Carl Roth, Karlsruhe, Germany, #6773.2) and 2 volumes of 95% (vol/vol) ethanol (Carl Roth, Karlsruhe, Germany, #K928.8), and incubated overnight at −80°C. On the following day, the samples were centrifuged at 25,000 × *g* for 5 min, and the pellet was washed 2 times with 70% (vol/vol) ethanol. After the final wash, the ethanol was removed, and the pellet was air dried and resuspended with ultrapure water, placed in a sonicating bath for 20 min, and stored at −20°C for further experiments.

Lipooligosaccharide (LOS) samples diluted 1:10 in Laemmli buffer were separated on a urea polyacrylamide separating gel (15% polyacrylamide [Carl Roth, Karlsruhe, Germany, #3029.1], 4 M urea [Sigma Aldrich, Darmstadt, Germany, #U5128], 0.375 M Tris/HCl, pH 8.8 [Carl Roth, Karlsruhe, Germany, #4855.2], 0.05% [vol/vol] ammonium persulfate [Sigma Aldrich, Darmstadt, Germany, #A3678], 0.1% [vol/vol] TEMED [Sigma Aldrich, Darmstadt, Germany, #T9281]) and a stacking gel (4.3% polyacrylamide [Carl Roth, Karlsruhe, Germany, #3029.1], 0.12 M Tris/HCl, pH 6.8 [Carl Roth, Karlsruhe, Germany, #4855.2], 0.1% [vol/vol] SDS [Carl Roth, Karlsruhe, Germany, #CN30.3], 0.05% [vol/vol] ammonium persulfate, and 0.1% [vol/vol] TEMED). After the run, the gel was washed with ultrapure water and fixed overnight in a 30% (vol/vol) ethanol (Carl Roth, Karlsruhe, Germany, #5054.4) and 10% (vol/vol) acetic acid (Carl Roth, Karlsruhe, Germany, #3738.5) solution. For the staining of the gel, the Pierce Silver staining kit (Thermo Fisher Scientific, Massachusetts, USA, #24612) was used following the manufacturer’s instructions.

### Barrier integrity of corneal epithelium models

To test the tissue integrity of the human cornea models upon infection, we measured the permeability to 4 kDa fluorescein isothiocyanate (FITC)-Dextran (Sigma Aldrich, Darmstadt, Germany, #46944). For this, 0.25 g/mL FITC-Dextran was dissolved in Dulbecco’s Modified Eagle Medium (DMEM) (Sigma Aldrich, Darmstadt, Germany, #D6429) and sterile filtered. The medium on the basolateral side of the insert was removed and replaced with 1 mL of DMEM, while 300 µL of the FITC-dextran solution was added to the apical side. The models were incubated for 30 min (37°C and 5% CO_2_), and 200 µL from the basolateral side was transferred to a black clear-bottom 96-well plate (Corning, New York, USA, #3603). The fluorescence was analyzed using a TECAN reader (490 nm absorption and 525 nm emission), and the results were normalized to the values measured from an empty cell culture insert.

### Cytotoxicity

Cytotoxicity was assessed by measuring the lactate dehydrogenase (LDH) activity in the cell supernatant using the Cytotoxicity Detection Kit PLUS (Roche, Basel, Switzerland, #4744926001). The assay was performed according to the manufacturer’s instructions, using the medium from the basolateral side of the models.

### Bacterial adherence

At different time points, the medium was removed from the basolateral side of the cell culture insert, and, using an 8 mm biopsy puncher, the membrane was removed and placed into an Eppendorf tube containing 300 µL of 1% (wt/vol) saponin solution (Sigma Aldrich, Darmstadt, Germany, #47036), and the tubes were vortexed for 1 min. After 30 min of incubation, serial dilutions were made and plated on GC agar plates. The CFU/mL was determined after 48 h of incubation at 37°C with 5% CO_2_.

### Cytokine measurements

Seventy-two hours after infection, the medium was collected from the basolateral side of the cell culture insert and analyzed using a personalized LegendPlex cytokine panel assay kit (BioLegend, San Diego, USA) to detect and measure the concentration of IL-1β, tumor necrosis factor (TNF)-α, IL-6, IL-8, IL-10, and Monocyte Chemoattractant Protein (MCP)-1. The assay was performed according to the manufacturer’s instructions, and the results were analyzed using the BioLegend software.

For the drug treatment experiments, the concentration of secreted IL-8 was measured in the medium collected from the basolateral compartment of the models infected with *N. gonorrhoeae* MS11 N159 and simultaneously treated with 40 µM trifluoperazine (SimTr). For this, the Human IL-8 DuoSet ELISA kit (R&D Systems, Minneapolis, USA, #DY208) was used, and the analysis was performed according to the manufacturer’s instructions.

### Immunofluorescence staining and imaging

At different time points, the medium was removed from the basolateral side of the cell culture inserts, and the infected corneal epithelium models were fixed with 4% paraformaldehyde (Morphisto, #11462.01000) for 2 h at room temperature, followed by washing three times with PBS and blocking with 3% (wt/vol) bovine serum albumin (Carl Roth, Karlsruhe, Germany, #8076.3) and 0.01% Triton X (Carl Roth, Karlsruhe, Germany, #3051.4) in PBS for 1 h. The models were then incubated with primary antibody against *N. gonorrhoeae* (United States Biological, Salem, USA, #N0600-02) at 4°C overnight. This was followed by incubation with a fluorophore-coupled secondary antibody (Alexa Fluor 488, Thermo Fisher Scientific, Massachusetts, USA, #A-11008), Phalloidin-Alexa Fluor 555 (Invitrogen/Thermo Fisher Scientific, Massachusetts, USA, #A34055), and DAPI (Sigma Aldrich, Darmstadt, Germany, #D9542). The samples were mounted using Mowiol (Carl Roth, Karlsruhe, Germany, #0713.2). For imaging, Z-stacks were made using a Leica STELLARIS 5 confocal imaging platform (Leica Microsystems, Wetzlar, Germany), and the images were processed using FIJI (13).

### Scanning electron microscopy

After 72 h of infection, the membranes of the cell culture inserts were carefully removed with an 8 mm biopsy puncher and were fixed with 6.5% glutaraldehyde at 4°C overnight. Subsequent sample preparation was done as previously described (14), and imaging was performed using a JEOL JSM-7500F microscope.

### Drug assays

We applied two different approaches to study the effects of trifluoperazine on pilus retraction. For the first approach, post-infection treatment (PostTr), the corneal epithelium models were infected with *N. gonorrhoeae* MS11 N159 for 1 h, as described previously in this study. The models were washed with PBS to remove the non-adherent bacteria and were then treated with 40 µM trifluoperazine (Sigma Aldrich, Darmstadt, Germany, #T8516) for 1 h. After this time, the models were washed with PBS and were left under the airlift condition for another 72 h. For the second approach, the simultaneous addition of bacteria and drug (SimTr), the models were infected with *N. gonorrhoeae* MS11 N159 as described and treated at the same time with 40 µM trifluoperazine. One hour later, the models were washed with PBS and left under the airlift condition for a further 72 h. Bacterial adherence was then measured as already described in this study.

### Bacterial growth curves

*N. gonorrhoeae* strains were grown for 24 h on GC agar plates. After this time, they were re-streaked onto a fresh GC agar plate for 16 h before the experiment. The bacteria were collected with a sterile cotton swab and diluted in 1 mL PPM+ medium (15 g/L proteose peptone [Becton Dickinson, New Jersey, USA, #211693], 5 g/L sodium chloride [VWR, Radnor, USA, #27810.364], 0.5 g/L soluble starch [Sigma-Aldrich, Darmstadt, Germany, #33615], 1 g/L potassium dihydrogen phosphate [Carl Roth, Karlsruhe, Germany, #3904.1], and 4 g/L dipotassium hydrogen phosphate [Carl Roth, Karlsruhe, Germany, #P749.3], supplemented with 1% [vol/vol] vitamin mix and 0.5% [vol/vol] sodium hydrogen carbonate [VWR, Radnor, USA, #27775.293]). The OD_550_ was measured and adjusted to 0.3 in 20 mL PPM+ medium and grown at 37°C with constant shaking (130 rpm) until they reached an OD_550_ of at least 0.4 (exponential phase). Then they were diluted to an OD_550_ of 0.1, with 40 µM trifluoperazine added to the corresponding cultures. The OD_550_ was measured every hour for 5 h.

### Bactericidal assay

To test the bactericidal effect of different concentrations of trifluoperazine in the two approaches (PostTr and SimTr), *N. gonorrhoeae* MS11 N159 were grown on GC agar plates for 24 h and then re-streaked onto a fresh plate, where they grew for approximately 16 h. The bacteria were collected with a sterile cotton swab, resuspended in 1 mL DMEM F12 medium, and the OD_550_ was measured. The volume needed to infect the corneal epithelium models with an MOI of 20 was calculated, and the solutions were prepared accordingly, with the different concentrations of trifluoperazine (10, 20, 30, 40, and 50 µM) and gentamicin (150 µg/mL) (Gibco, Thermo Fisher Scientific, Massachusetts, USA, #15710-049). A volume of 100 µL of each solution was added to a flat-bottom 96-well plate.

For the first approach (PostTr), the plate containing bacteria only was incubated for 1 h at 37°C with 5% CO_2_; after this time, trifluoperazine and gentamicin were added to the corresponding wells for 1 h of treatment. For the second approach (SimTr), the plate containing the bacterial suspension with different concentrations of trifluoperazine and gentamicin was incubated for 1 h at 37°C with 5% CO_2_. After incubation, serial dilutions were made and plated onto GC agar plates. Colonies were counted after 48 h to calculate the CFU/mL.

### Aggregation assay

To analyze the effect of trifluoperazine on the disaggregation of microcolonies, *N. gonorrhoeae* MS11 N159 were grown for 24 h on a GC agar plate, re-streaked onto a fresh GC agar plate, and cultivated for approximately 16 h. The bacteria were collected with a sterile cotton swab, diluted in 1 mL PPM+ medium, and the OD_550_ was measured. The OD was adjusted to 0.3 in 15 mL PPM+ medium, and bacteria were grown at 37°C with constant shaking (130 rpm) until they reached an OD_550_ of approximately 0.4. The bacterial culture was diluted with DMEM F12 medium to an OD of 0.1. A 500 µL of this suspension was distributed into a 24-well plate and incubated (37°C, 5% CO_2_) for 2 h. After this time, the bacteria were treated with increasing concentrations of trifluoperazine (10, 20, 30, 40, and 50 µM) and with gentamicin (150 µg/mL) for 1 h. Before and after the treatment, three images per well were taken using a Leica DMIRB microscope (Leica Microsystems, Wetzlar, Germany), and the area of the microcolonies was calculated using FIJI (13).

### Statistical methods

Statistical analyses were performed on at least three independent biological replicates using two-way ANOVA and Tukey’s multiple comparison tests, with the help of the GraphPad Prism software version 10.4.2.

## RESULTS

### Type IV pilus plays a crucial role in the adherence of *N. gonorrhoeae* to the corneal epithelium

**Fig 1:**
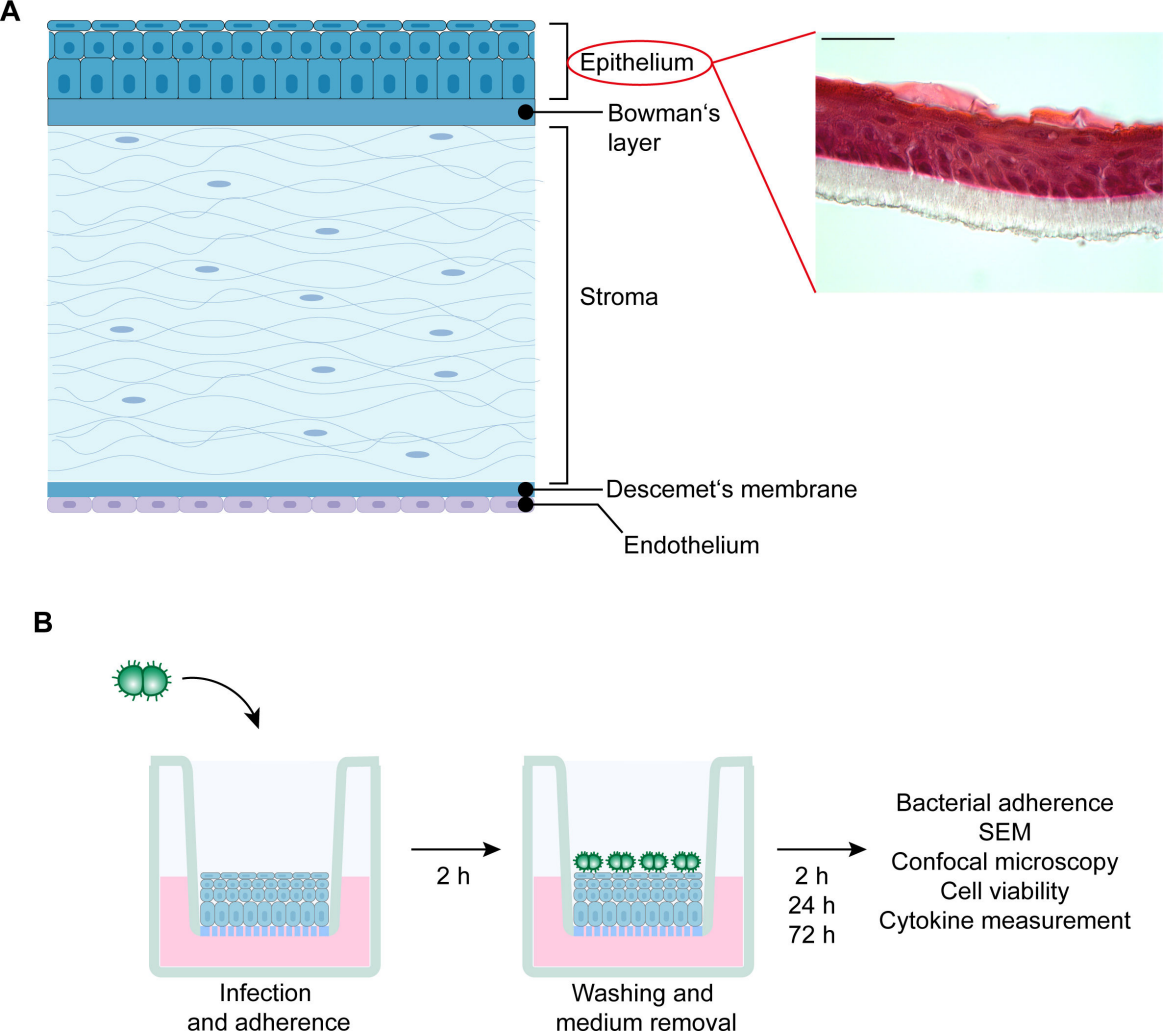
Schematic representation of the corneal epithelium structure and study design. (**A**) The scheme of the cornea structure (on the left side) in comparison to the corneal epithelium model used in this study. The corneal tissue model was fixed, embedded in paraffin, sectioned into 7 µm-thick sections, and stained using hematoxylin and eosin. Scale bar is 50 µm. (**B**) Corneal tissue models are infected from the apical side with the different *N. gonorrhoeae* MS11 derivative strains for 2 h, followed by washing with PBS and further incubation under airlift conditions. Different parameters are analyzed at different time points.

To study the role of the type IV pilus of *N. gonorrhoeae* in adherence to the intact corneal epithelium, we used a multilayer model made of immortalized human-derived corneal epithelial cell lines (hTCEpi) seeded onto polycarbonate membrane cell culture inserts. These models show corneal differentiation traits, and their architecture resembles the native tissue (Fig. 1A) (10). We allowed bacteria to interact with the tissue for 2 h before washing and removing those that did not adhere. Further analyses of the models were then performed at different infection times, starting directly after the wash (2 h time point) and finishing 72 h later (Fig. 1B).

**Fig 2:**
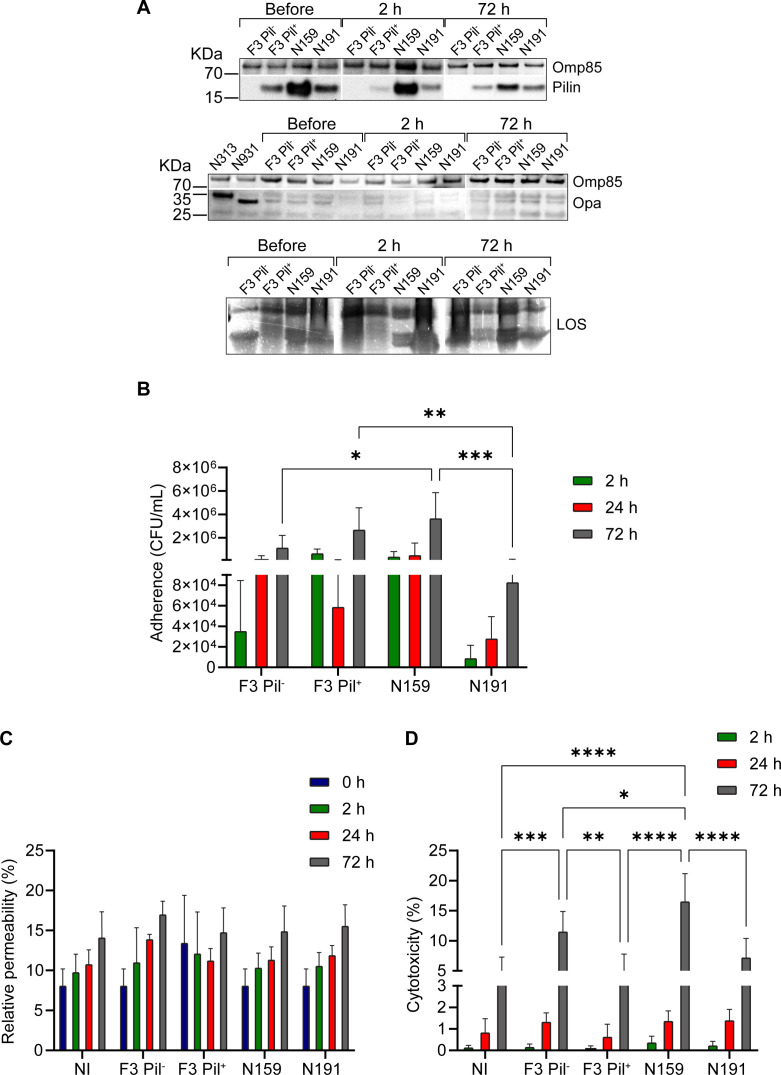
Impact of pilus expression on bacterial adherence, corneal tissue model barrier integrity, and cytotoxicity. (**A**) The expression of Pilin, Opa, and LOS in different *N. gonorrhoeae* MS11 derivative strains was analyzed by polyacrylamide gel electrophoresis in bacteria used to infect tissue models (before), in bacteria that did not adhere to the tissue models after 2 h of infection (2 h), and in bacteria that could be isolated from the tissue models after 72 h of infection (72 h). For immunoblotting, antibodies against Pilin, Omp85 (outer membrane protein assembly factor BamA), and Opa were used. N313 and N931 bacteria, expressing Opa^57^ and Opa^50^, respectively, served as controls. LOS was visualized by silver staining. (**B**) To determine the number of adherent bacteria, corneal tissue models were solubilized with 1% saponin for 30 min. Serial dilutions were plated on GC agar to calculate the CFU/mL. (**C**) The corneal epithelium barrier integrity was assessed before (0 h) and at different time points of infection by measuring the diffusion of a FITC-dextran solution from the apical to the basolateral compartment of the models. The values were normalized to the values of an empty cell insert. (**D**) Cytotoxicity during different time points of infection was measured as the percentage of LDH released into the medium collected from the basolateral side of the corneal epithelium models. All graphs represent mean values ± SD from three independent replicates. Statistical analysis was done using two-way ANOVA and Tukey’s multiple comparison tests. *P ≤ 0.05, **P ≤ 0.01, ***P ≤ 0.001, and ****P ≤ 0.0001.

We infected the models with four different *N. gonorrhoeae* strains derived from MS11, which exhibit different pilus phenotypes (Fig. 2A). To assess these differences, we first performed whole-genome sequencing of each strain and analyzed the regions encoding for important proteins like PilE, PilC, Opa proteins, and RecA (Table 1). We observed that one of the two *pilE* genes in the F3 Pil− strain was not present and the other one was partially deleted, resulting in no expression of pilin, which was confirmed by western blot of bacteria before and after 2 h of infection, as well as in bacteria collected from the models after 72 h of infection (Fig. 2A; Fig. S1). This gene was intact in F3 Pil+, N159, and N191; however, there was a noticeably higher expression of the pilus in N159 (Fig. 2A). In addition, the *recA* gene in N159 is inactivated due to a large insertion, which drastically reduces phase variation of the pilus. N191, although possessing an intact *pilE* gene, has mutations in both *pilC* genes, which strongly diminished the adherence of these bacteria (Table 1). Opa protein expression was low in all the strains used in comparison to the N313 and N931 control strains that express Opa^57^ and Opa^50^ (12), and this did not change during infection. Comparison between different mutants before and after infection showed similar content of LOS with slight chemotype variation (Fig. 2A).

**TABLE 1:** Comparison of relevant genes involved in bacterial adherence and invasion between different *N. gonorrhoeae* MS11 derivative strains and the reference genome of *N. gonorrhoeae* MS11 (NCBI, CP003909) according to the information obtained by whole-genome sequencing^*a*^

Gene	Gene ID	Function	Strain			
			MS11 F3 Pil−	MS11 F3 Pil+	MS11 N159	MS11 N191
*pilE*	NGFG_01821	Major pilin subunit	Deletions	NC	NC	NC
	NGFG_01819		Not present	NC	NC	NC
*pilS*	NGFG_02405	Antigenic variation of *pilE*	NC	NC	NC	NC
	NGFG_02431		NC	NC	NC	NC
	NGFG_02481		NC	NC	NC	NC
	NGFG_02482		NC	NC	NC	NC
	NGFG_02484		NC	NC	NC	NC
*pilX*	NGFG_00609	GC aggregation	NC	NC	NC	NC
*pilG*	NGFG_02119	Pilus assembly	NC	NC	NC	NC
*pilC1*	NGFG_01977	Pilus tip adhesin	NC	NC	NC	1,158 bp insertion
*pilC2*	NGFG_00188		NC	NC	NC	Not present
*opa*	NGFG_02238	Mediate adhesion and invasion (epithelial cells, neutrophils, T and B cells)	NC	NC	NC	NC
	NGFG_02258		NC	NC	NC	NC
	NGFG_02259		NC	NC	NC	NC
	NGFG_02330		NC	NC	NC	NC
	NGFG_02337		NC	NC	NC	NC
	NGFG_02351		NC	NC	NC	NC
	NGFG_02406		NC	Shorter sequence	Shorter sequence	Shorter sequence
	NGFG_02432		NC	NC	NC	NC
	NGFG_02435		NC	NC	NC	NC
	NGFG_02467		NC	NC	NC	NC
	NGFG_02483		NC	Not present	Not present	Not present
*recA*	NGFG_01045	Pilus antigenic variation	NC	NC	518 bp insertion	NC
*porB_IB_*	NGFG_01725	Outer membrane porin	NC	NC	NC	NC

^*a*^ NC, no changes in the sequence compared to the reference genome sequence.

We assessed bacterial adherence, cytotoxicity, and tissue permeability during infection. N159 strain showed the highest adherence at all time points (2, 24, and 72 h). The adherence capacity of F3 Pil+ was the second highest (Fig. 2B). This strain resembles the wild-type MS11 genotype and contains the intact *recA* gene, so the pilus undergoes phase variation at a much higher rate compared to N159; this was reflected in the fact that the pilus expression in the bacteria collected after 2 h of infection was reduced (Fig. 2A), indicating that non-adherent bacteria were less piliated. F3 Pil− strain showed a somewhat reduced adherence capacity, as expected, due to the lack of PilE expression (Fig. 2A and B), especially at 2 h. Lastly, the N191 strain showed the lowest adherence to the corneal epithelium, emphasizing the importance of PilC in the adhesion to the target tissue (6) (Fig. 2B).

The mature models at the time of infection have a relative permeability of 8%, as measured by the FITC-dextran assay. The permeability increased over time to up to 15% in both uninfected and infected models, indicating that this was probably caused by the aging of the tissue models and not by infection (Fig. 2C).

The cytotoxicity upon infection was measured in relation to the concentration of LDH released into the medium of the basolateral compartment. This is connected to cell lysis, usually associated with apoptosis or necrosis. We observed an increase in cytotoxicity with time, showing the highest percentage in the models infected with the N159 strain for 72 h (17%). Interestingly, the F3 Pil− strain showed the second highest cytotoxicity, followed by F3 Pil+ and N191, for which the values were comparable to those obtained for the non-infected models (Fig. 2D).

In conclusion, adherence and cytotoxicity mostly correspond to the ability of bacteria to adhere to the corneal tissue models with the help of the pilus. Relatively high adherence and cytotoxicity of F3 Pil− strain indicate, however, that bacteria can use other mechanisms besides pili to efficiently adhere to the corneal tissue.

### *N. gonorrhoeae* forms microcolonies on the surface of corneal tissue models, depending on the presence of the pilus

Another important role of the type IV pilus in *N. gonorrhoeae* is the formation of microcolonies. They allow GC to survive longer in the target tissue in the presence of some antibiotics, due to their limited diffusion within the microcolony (15).

**Fig 3:**
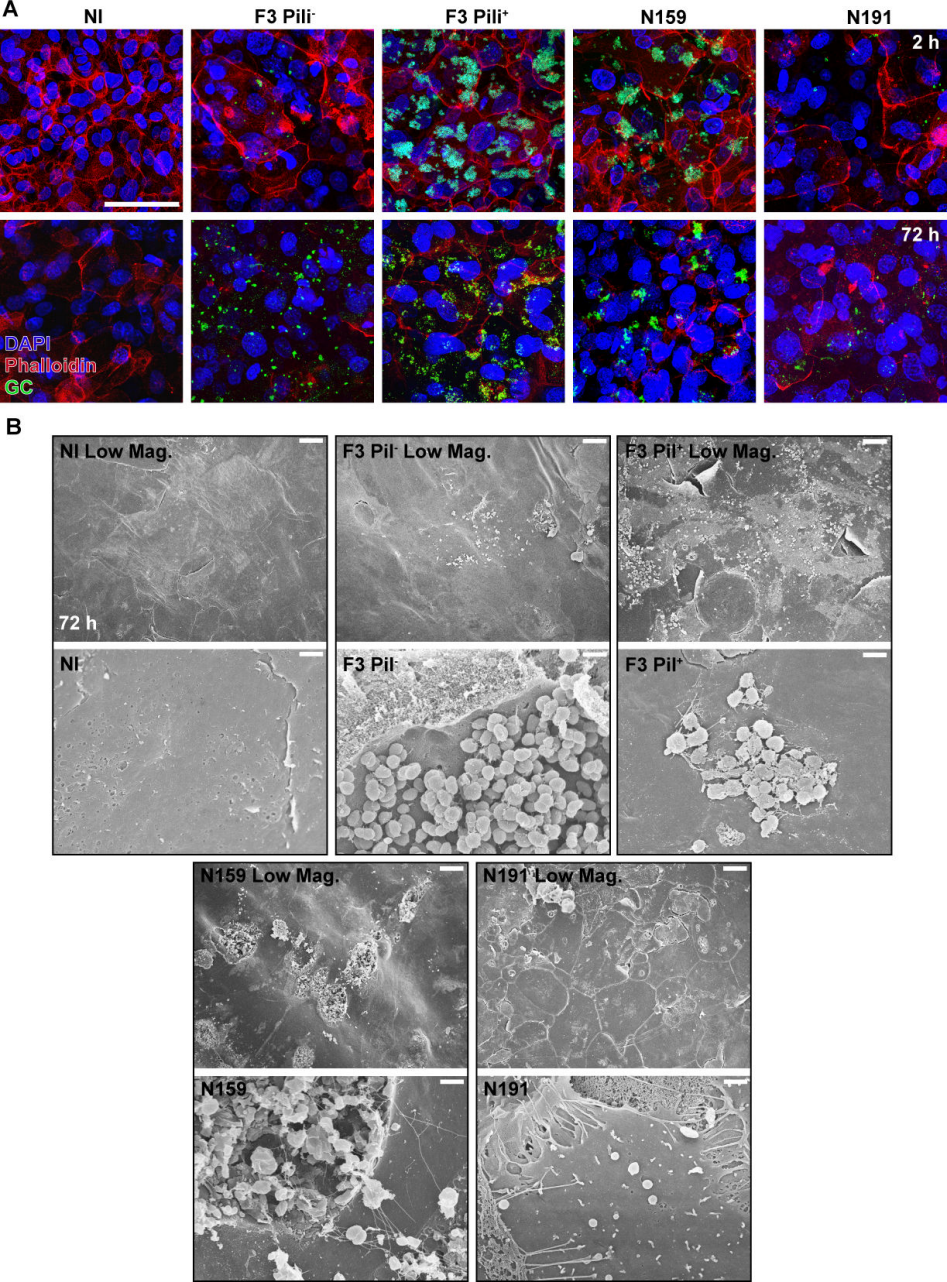
Pilus-expressing *N. gonorrhoeae* MS11 derivative strains form microcolonies on the surface of corneal tissue models. (**A**) The models were fixed 2 and 72 h post-infection and analyzed by immunofluorescence and confocal microscopy, using DAPI (blue), Phalloidin-Alexa Fluor 555 (red), and the primary antibody against *N. gonorrhoeae*/Alexa Fluor 488-coupled secondary antibody (green). The images were made using the Leica Stellaris 5 confocal imaging platform. Scale bar is 50 µm. (**B**) Corneal tissue models infected with different *N. gonorrhoeae* MS11 derivative strains were fixed after 72 h of infection and analyzed by scanning electron microscopy (SEM). Scale bars are 10 µm for low magnification (Low Mag.) and 1 µm for other images.

We observed large microcolonies already after 2 h of infection for the piliated strains F3 Pil+ and N159. These microcolonies were distributed over the whole surface of the corneal epithelium models. In contrast, F3 Pil− and N191 bacteria were present on the models as single bacteria or in much smaller aggregates, visible only after 72 h of infection (Fig. 3A).

Scanning electron microscopy (SEM) of the infected tissue models 72 h post-infection revealed that F3 Pil+ and N159 microcolonies led to tissue damage, with pits forming underneath bacterial aggregates attached and connected through long filaments, most likely representing pili. Such filaments were absent in F3 Pil−, though we observed occasional accumulations of these bacteria resembling a biofilm. N191 was hard to detect in SEM images and was found only as single diplococci attached to the cell surface. Interestingly, pili filaments of N159 appeared much longer and more abundant than those of F3 Pil+ (Fig. 3B).

Microscopy shows, therefore, that only piliated bacteria form large microcolonies, which lead to tissue damage. Consequently, the high cytotoxicity observed for the N159 strain corresponds to the most pronounced tissue damage visible on the SEM images.

### Corneal epithelial cells produce different cytokines and chemokines upon infection with *N. gonorrhoeae*

Previous studies have shown that bacteria induce the secretion of different cytokines by the mucosal epithelial cells, which are the first responders to infection. The secreted cytokines regulate other cell types, recruiting immune cells to the site of infection. The degree of this response is dependent on the cell type, as well as on the pathogen and its virulence factors (16).

**Fig 4:**
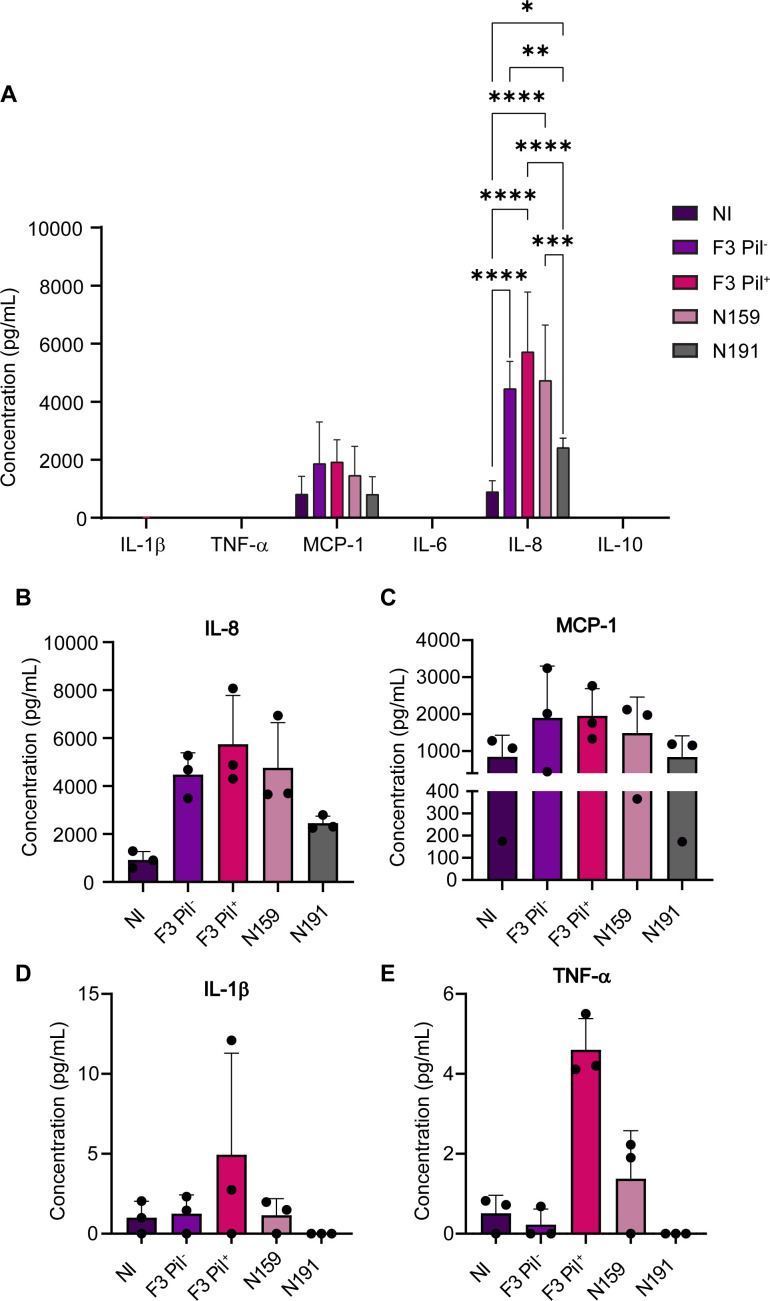
Infection of corneal epithelium models *N. gonorrhoeae* MS11 leads to an increase in IL-8 and TNF-α secretion. (**A–E**) Different cytokines and chemokines secreted by the corneal epithelial cells were measured in the medium collected from the basolateral side of the tissue models after 72 h of infection using a personalized LegendPlex cytokine panel to assess the secretion of IL-8 (**B**), MCP-1 (**C**), IL-1β (**D**), and TNF-α (**E**). The graphs represent the mean values ± SD from three independent replicates. Statistical analysis was performed using two-way ANOVA and Tukey’s multiple comparison tests. *P ≤ 0.05, **P ≤ 0.01, ***P ≤ 0.001, and ****P ≤ 0.0001.

We evaluated the secretion of different cytokines and chemokines after 72 h of infection with the four strains we used. Interleukin (IL)-8 was the most secreted cytokine, followed by MCP-1, IL-1β, and TNF-α, whereas IL-6 and IL-10 were not detectable or present in very low amounts (below 3 pg/mL) (Fig. 4A). The main function of IL-8 is the recruitment of neutrophils in response to tissue damage caused by infection or injury (17). Whereas infection generally led to a significant increase in IL-8 production, the models infected with the piliated strains F3 Pil+ and N159 showed the highest secretion of IL-8, while the non-adherent N191 showed the lowest. The differences in IL-8 production between the models infected with F3 Pil−, F3 Pil+, or N159 were not statistically significant (Fig. 4A and B). The high secretion of IL-8 correlates in part with the observed adherence and tissue damage (Fig. 2B and D).

The secretion of MCP-1, another chemokine responsible for the recruitment of monocytes and macrophages, was also observed in all infected models, although without statistically significant differences among them and non-infected models. However, similar to the IL-8 secretion, F3 Pil−, F3 Pil+, and N159 strains showed the highest values, while N191 showed the lowest (Fig. 4C). Regarding IL-1β and TNF-α, we detected amounts less than 10 pg/mL in the infected tissue models with F3 Pil+, N159, and F3 Pil−. For the N191 strain, values were under the detection limit (Fig. 4D and E).

The infection, therefore, induces the secretion of high amounts of IL-8 that correlate with the ability of bacteria to adhere to the tissue and are the highest for the strains that express a functional pilus.

### Trifluoperazine decreases bacterial adherence and secretion of IL-8

We next explored whether our multilayer corneal epithelium infection models are suitable for drug testing. For this, we used trifluoperazine, a small molecule that belongs to the group of phenothiazines and is currently used as an antipsychotic agent. Previously, trifluoperazine was shown to have antimicrobial properties, especially against bacteria that express the type IV pilus (18). In previous studies, trifluoperazine disrupted pilus-dependent functions, such as twitching motility, aggregation, and adherence to inert surfaces or cell surfaces in *N. meningitidis* (19). In our study, we assessed the adherence to the corneal epithelium tissue models and the IL-8 secretion after treatment with trifluoperazine, using a highly piliated N159 *N. gonorrhoeae* MS11 derivative.

**Fig 5:**
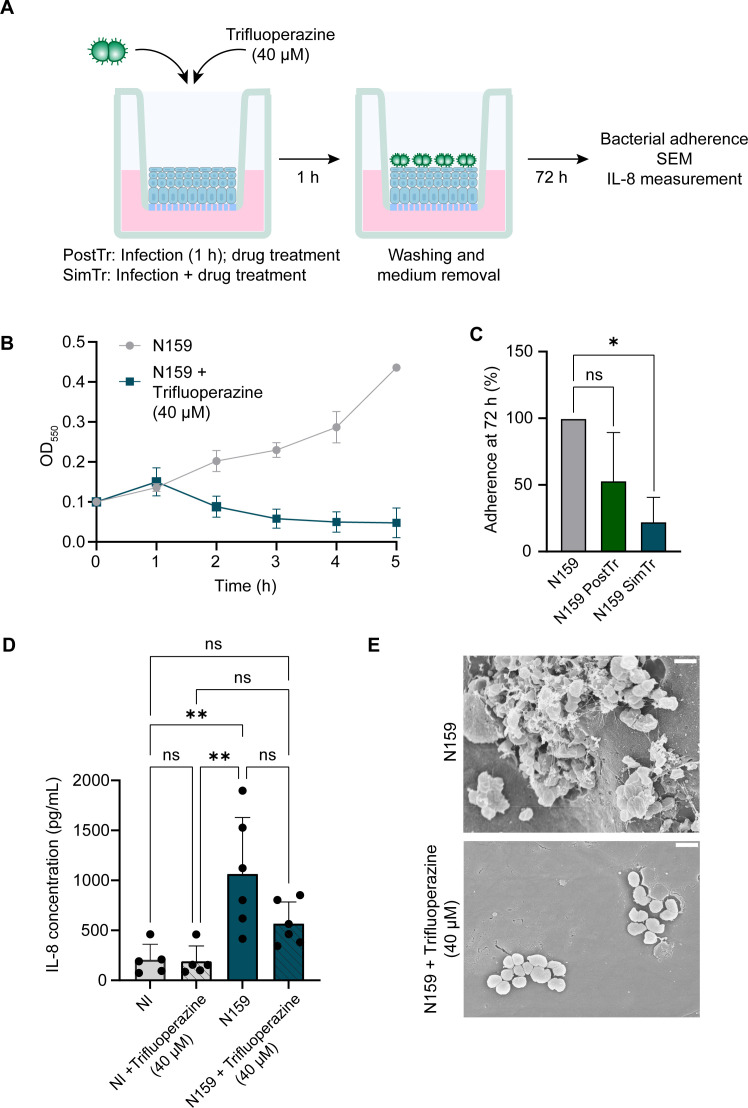
Trifluoperazine decreases bacterial adherence to the corneal epithelium models in the early stages of infection. (**A**) Schematic representation of two different approaches to test the effect of trifluoperazine. In the PostTr approach, models were first infected with N159 bacteria for 1 h, washed, and treated with 40 µM trifluoperazine for 1 h, followed by another wash and incubation for 72 h under airlift conditions. For the SimTr approach, the bacteria were treated with 40 µM trifluoperazine simultaneously with infection for 1 h, after which the models were washed and kept for 72 h under airlift conditions. (**B**) To measure the growth of N159 bacteria without and with 40 µM trifluoperazine, bacteria were grown in the PPM+ medium until they reached an OD_550_ = 0.4, diluted to an OD_550_ = 0.1, and 40 µM trifluoperazine was added to one culture. OD_550_ was measured every hour for 5 h. The graph represents mean values ± SD of at least three independent replicates. (**C**) Models were prepared as described in panel **A** and solubilized with 1% saponin solution. The CFU/mL was calculated from the number of colonies growing after plating serial dilutions of the suspension on GC agar plates. The results were normalized to the non-treated models. The graph represents mean values ± SD of at least three independent replicates. (**D**) The concentration of IL-8 was measured in the basolateral medium of the non-infected (NI) and N159-infected corneal tissue models, with and without 40 µM trifluoperazine, using ELISA. The graph shows the mean value of at least three independent replicates. Statistical analysis was performed using two-way ANOVA and Tukey’s multiple comparison tests. *P ≤ 0.05, **P ≤ 0.01; ns, not significant. (**E**) Scanning electron microscopy images of the corneal epithelium models infected with N159 with and without the 1 h treatment with 40 µM trifluoperazine. Scale bar is 1 µm.

In the first approach, we infected cornea tissue models with the N159 strain for 1 h, administered trifluoperazine (40 µM) for another hour, and then removed the medium and the drug, allowing the infection to proceed for another 72 h (post-treatment [PostTr]). In the second approach, bacteria and the drug were simultaneously added to the models for 1 h, after which they were removed by washing, followed by an additional 72 h of infection (simultaneous treatment [SimTr]) (Fig. 5A).

To control for the bactericidal effects of trifluoperazine, we monitored bacterial growth and survival in the presence of the drug. Starting from the second hour, trifluoperazine strongly affected the growth of N159 (Fig. 4B). However, analysis of bacterial survival after 1 h of treatment with different trifluoperazine concentrations in the PostTr and SimTr setups revealed interesting differences. The drug had no significant effect on bacterial survival when bacteria were first allowed to form microcolonies for 1 h (Fig. S2A). On the other hand, we observed a concentration-dependent decrease in the number of surviving bacteria when the drug was added directly to the bacterial suspension for 1 h, without previous incubation that would let bacteria aggregate (Fig. S2B). In both cases, treatment with gentamicin completely eradicated the bacteria.

Additionally, we studied the effect that trifluoperazine has on *N. gonorrhoeae* microcolonies. We incubated the suspension of N159 bacteria in a tissue culture plate for 2 h, which was sufficient for microcolony formation, and then added trifluoperazine in increasing concentrations, measuring the surface area of the microcolonies before and after 1 h of drug treatment. The drug significantly diminished the size of the microcolonies (Fig. S2C), though this did not correlate with the bactericidal effect (Fig. S2A). Gentamicin, on the other hand, did not affect bacterial microcolony size even though no bacteria survived the treatment (Fig. S2C).

In agreement with these observations, we observed no significant difference in N159 adherence to the corneal tissue models in the PostTr setup, whereas the simultaneous addition of trifluoperazine significantly diminished the number of adherent bacteria (Fig. 5C). Accordingly, the drug treatment led to a reduction in IL-8 secretion in the infected corneal epithelium models (Fig. 5D). In addition, the SEM showed that, after the addition of trifluoperazine, N159 microcolonies on the surface of the tissue models were reduced to only a few bacteria, with no visible pilus present (Fig. 5E).

Corneal epithelium tissue models, therefore, represent a reliable tool to test drugs that affect bacterial pilus formation and adherence. One of these drugs is trifluoperazine, which can reduce gonococcal infectivity by affecting pilus formation.

## DISCUSSION

In this work, we show the application of a multilayer corneal epithelium tissue model for the study of *N. gonorrhoeae* infection. These models were developed using an immortalized human corneal epithelial cell line (hTCEpi) grown on a polycarbonate membrane scaffold. hTCEpi expresses a human telomerase reverse transcriptase, leading to the activation of telomerase, which prevents the shortening of telomeres and telomere-dependent senescence in the cells without altering their differentiation capacity (20). Under airlift conditions, these models exhibit differentiation markers of the corneal epithelium and natural desquamation comparable to native corneal tissue (10). However, for infection studies, additional parameters like tissue permeability and integrity are also important. Our multilayer corneal epithelium models have a permeability of 8% and transepithelial electrical resistance values of 327 Ω*cm^2^ on day 7 after introducing airlift conditions, which correlates with values measured for human cornea (200 Ω*cm^2^) (21), making them suitable to be used as infection models.

One of the most important roles of the type IV pilus of *N. gonorrhoeae* is to enable bacteria to adhere to tissues and form microcolonies (22, 23). In our experiments using hTCEpi-based corneal tissue models, we observed that the adherence of the piliated F3 Pil+ and N159 strains was indeed the highest, as expected, compared to lower adherence of F3 Pil− and especially N191 (Fig. 2B). N191 strain expresses pilus and has an intact *recA* gene, but has both *pilC* genes mutated or deleted. These mutations in *pilC*, which encodes for the subunit located at the tip of the pilus, described as the adhesin that allows the contact of the bacteria with the target tissue and other bacteria, are responsible for the reduced adherence capacity of this strain, even though PilE protein is expressed (Fig. 2A) (6). In our infection model, we could faithfully reproduce the effect of the PilC defect and consistently observe low adherence of N191 bacteria.

The differences in adhesion between piliated and non-piliated/non-adherent bacteria were particularly noticeable at the earliest time point of 2 h. At later time points, the number of adherent bacteria increased for all strains (Fig. 2B), probably due to the multiplication of already-attached GC (Fig. 3A). Interestingly, F3 Pil− showed better adherence than the N191 strain, indicating that the presence of a non-functional pilus might interfere with the tissue contact. Both strains still interacted with the tissue, however, possibly through Opa proteins (24) or LOS, since we did not specifically select Opa-negative bacterial colonies for infection, and bacteria still contained low amounts of Opa (Fig. 2A). An earlier report showed that non-piliated bacteria did not adhere to human corneal explants, and that the adherence was independent of the Opa status (8). However, in that study, bacteria were incubated with the tissue for 1 h, whereas in our experiments, the incubation time was 2 h. It is possible that longer incubation allowed bacteria to interact with the tissue using Opa proteins, and that the differences in adherence between piliated and non-piliated bacteria would be more apparent at shorter incubation times.

The same earlier study reported thinning and exfoliation of the human cornea explants upon infection (8). *N. gonorrhoeae* was also shown to disrupt the apical junctions of polarized epithelial cells (25, 26) and induce epithelial cell exfoliation in cervical tissue explants (27). Contrary to this, we did not see major differences in tissue permeability changes over time between infected and non-infected hTCEpi cornea models that would be indicative of tissue thinning or exfoliation (Fig. 2C). The cytotoxicity also did not seem to strictly depend on the presence of the pilus but mostly correlated with the number of adherent bacteria (Fig. 2D). Greater robustness of cornea tissue models in infection scenarios might be due to the differences between tissue explants and models, for example, because of the residual immune cells in corneal explants that mediate a tissue-damaging immune response.

The formation of microcolonies is important for the survival of GC in the host because it facilitates invasion and reduces the effectiveness of antibiotics, as they are less able to diffuse within the microcolony. In our study, we observed tissue damage and formation of pits under the microcolonies formed by highly piliated N159. This is reminiscent of the observations made using human cornea explants (8). Tissue damage caused by GC attachment could be related to cornea perforation, one of the most severe complications in patients with untreated eye infections caused by *N. gonorrhoeae* (28, 29). Likewise, SEM images at 72 h revealed that F3 Pil− forms occasional biofilm-like patches on the surface of the models. No pilus was visible, but there was an apparent tissue destruction beneath these patches (Fig. 3B), which could explain the high cytotoxicity measured for models infected with this strain (Fig. 2D). Interestingly, however, the lowest percentage of cytotoxicity was seen after F3 Pil+ infection and not after N191 infection, suggesting that the cell death caused by GC in the corneal epithelium tissue is not exclusively pilus-dependent, and the role of other virulence factors is also relevant.

We also measured the concentration of different cytokines and chemokines secreted upon infection by the corneal epithelial cells. The pattern of cytokines depends on the tissue in question. In T-cells, the pilus-dependent adherence of *N. gonorrhoeae* induces the activation and proliferation of these cells and the production of anti-inflammatory IL-10 (30). In mature macrophages incubated with piliated GC, the infection causes an increased secretion of the pro-inflammatory cytokines IL-6, TNF-α, GRO-α, MIP-1α, and RANTES, with no effect on IL-8 (31). In the male urethra (32), there is an increased expression of IL-8, IL-6, and TNF-α in urine before the start of GC infection symptoms, and in fallopian tubes, IL-1α, IL-1β, and TNF-α are upregulated (33). Our previous work using 3D models that mimicked endometrial and urethral tissue showed that urethral tissue models secreted high amounts of IL-8, IL-6, and TNF-α, whereas endometrial tissue models secreted significantly less of the same cytokines (12). There is little known, however, about the cytokine secretion of corneal epithelium upon GC infection. We observed an increase in IL-8 and TNF-αsecretion in infected cornea models, which correlated with the number of adherent bacteria. MCP-1 secretion was also increased, but this did not seem to be related to the infection. Only small amounts of IL-1β, and no IL-6 or IL-10, were produced upon infection (Fig. 4). These results point to a specific, pro-inflammatory response of corneal tissue to GC infection, mostly directed at the recruitment of neutrophils, which corresponds to the clinical picture of the disease.

Finally, we tested the effects of trifluoperazine on pilus-mediated adherence and infection using corneal tissue models. Trifluoperazine reduces bacterial piliation by affecting the establishment of the sodium gradient through alteration of the activity of the Na^+^-pumping NADH-ubiquinone oxidoreductase complex (19). This complex creates a Na^+^ gradient across the cell membrane using energy released by the oxidation of NADH and the reduction of quinone (34). Trifluoperazine was described to induce pilus retraction and dispersal of microcolonies in *N. meningitidis* (19). We demonstrated a similar effect on *N. gonorrhoeae* in liquid culture (Fig. S2). However, in tissue models, we observed that when we allowed piliated GC to adhere and form microcolonies on the tissue models for 1 h before the addition of trifluoperazine, the drug was not as efficient as when added simultaneously with bacteria (Fig. 5C). This is in agreement with the role of the pilus only in the initial attachment to the tissue, with adherence to host cells being mediated by other adhesins as the infection progresses.

Our study demonstrates for the first time the usefulness of hTCEpi-based tissue models for infection research, shedding light on several important aspects of the infection of the cornea with *N. gonorrhoeae* and the role of type IV pilus in this process. In comparison to the primary tissue in the form of explants, corneal epithelial tissue models are more accessible and show lower variability, while still faithfully mimicking the original tissue, making them suitable for high-throughput research and drug screening.

## Data Availability

Sequencing data related to this study are available and can be accessed from BioProject PRJNA1356842.

## SUPPLEMENTAL MATERIAL

Supplemental figures iai.00735-25-s0001.pdf Fig. S1 and S2.

ASM does not own the copyrights to Supplemental Material that may be linked to, or accessed through, an article. The authors have granted ASM a non-exclusive, world-wide license to publish the Supplemental Material files. Please contact the corresponding author directly for reuse.

## References

[1] Churchward CP, Snyder LAS. 2019. In vitro models of eye infection with Neisseria gonorrhoeae. Methods Mol Biol 1997:363–376. doi:10.1007/978-1-4939-9496-0_21 31119634

[2] Churchward CP, Alany RG, Kirk RS, Walker AJ, Snyder LAS. 2017. Prevention of ophthalmia neonatorum caused by Neisseria gonorrhoeae using a fatty acid-based formulation. mBio 8:e00534-17. doi:10.1128/mBio.00534-17 28743809 PMC5527305

[3] Craig L, Forest KT, Maier B. 2019. Type IV pili: dynamics, biophysics and functional consequences. Nat Rev Microbiol 17:429–440. doi:10.1038/s41579-019-0195-4 30988511

[4] Rudel T, van Putten JP, Gibbs CP, Haas R, Meyer TF. 1992. Interaction of two variable proteins (PilE and PilC) required for pilus-mediated adherence of Neisseria gonorrhoeae to human epithelial cells. Mol Microbiol 6:3439–3450. doi:10.1111/j.1365-2958.1992.tb02211.x 1362447

[5] Rudel T, Boxberger HJ, Meyer TF. 1995. Pilus biogenesis and epithelial cell adherence of Neisseria gonorrhoeae pilC double knock-out mutants. Mol Microbiol 17:1057–1071. doi:10.1111/j.1365-2958.1995.mmi_17061057.x 8594326

[6] Rudel T, Scheurerpflug I, Meyer TF. 1995. Neisseria PilC protein identified as type-4 pilus tip-located adhesin. Nature 373:357–359. doi:10.1038/373357a0 7830772

[7] Heydarian M, Rühl E, Rawal R, Kozjak-Pavlovic V. 2022. Tissue models for Neisseria gonorrhoeae research—from 2D to 3D. Front Cell Infect Microbiol 12. doi:10.3389/fcimb.2022.840122 PMC8873371 35223556

[8] Tjia KF, van Putten JP, Pels E, Zanen HC. 1988. The interaction between Neisseria gonorrhoeae and the human cornea in organ culture. An electron microscopic study. Graefes Arch Clin Exp Ophthalmol 226:341–345. doi:10.1007/BF02172964 3139497

[9] Lotz C, Kiesewetter L, Schmid FF, Hansmann J, Walles H, Groeber-Becker F. 2018. Replacing the Draize eye test: Impedance spectroscopy as a 3R method to discriminate between all GHS categories for eye irritation. Sci Rep 8:15049. doi:10.1038/s41598-018-33118-2 30301970 PMC6177390

[10] Robertson DM, Li L, Fisher S, Pearce VP, Shay JW, Wright WE, Cavanagh HD, Jester JV. 2005. Characterization of growth and differentiation in a telomerase-immortalized human corneal epithelial cell line. Invest Ophthalmol Vis Sci 46:470–478. doi:10.1167/iovs.04-0528 15671271

[11] Vohra M, Kumar S, Sohnen P, Kaur S, Swamynathan S, Hirose T, Kozmik Z, Swamynathan SK. 2025. Pard3 promotes corneal epithelial stratification and homeostasis by regulating apical-basal polarity, cytoskeletal organization and tight junction-mediated barrier function. Ocul Surf 37:201–215. doi:10.1016/j.jtos.2025.04.001 40188986 PMC13455037

[12] Heydarian M, Yang T, Schweinlin M, Steinke M, Walles H, Rudel T, Kozjak-Pavlovic V. 2019. Biomimetic human tissue model for long-term study of Neisseria gonorrhoeae infection. Front Microbiol 10:1740. doi:10.3389/fmicb.2019.01740 31417529 PMC6685398

[13] Schindelin J, Arganda-Carreras I, Frise E, Kaynig V, Longair M, Pietzsch T, Preibisch S, Rueden C, Saalfeld S, Schmid B, Tinevez J-Y, White DJ, Hartenstein V, Eliceiri K, Tomancak P, Cardona A. 2012. Fiji: an open-source platform for biological-image analysis. Nat Methods 9:676–682. doi:10.1038/nmeth.2019 22743772 PMC3855844

[14] Spiliotis M, Lechner S, Tappe D, Scheller C, Krohne G, Brehm K. 2008. Transient transfection of Echinococcus multilocularis primary cells and complete in vitro regeneration of metacestode vesicles. Int J Parasitol 38:1025–1039. doi:10.1016/j.ijpara.2007.11.002 18086473

[15] Tseng BS, Zhang W, Harrison JJ, Quach TP, Song JL, Penterman J, Singh PK, Chopp DL, Packman AI, Parsek MR. 2013. The extracellular matrix protects Pseudomonas aeruginosa biofilms by limiting the penetration of tobramycin. Environ Microbiol 15:2865–2878. doi:10.1111/1462-2920.12155 23751003 PMC4045617

[16] Hedges SR, Agace WW, Svanborg C. 1995. Epithelial cytokine responses and mucosal cytokine networks. Trends Microbiol 3:266–270. doi:10.1016/S0966-842X(00)88941-6 7551639

[17] Kobayashi Y. 2008. The role of chemokines in neutrophil biology. Front Biosci 13:2400–2407. doi:10.2741/2853 17981721

[18] Duménil G. 2019. Type IV pili as a therapeutic target. Trends Microbiol 27:658–661. doi:10.1016/j.tim.2019.05.005 31182345

[19] Denis K, Le Bris M, Le Guennec L, Barnier J-P, Faure C, Gouge A, Bouzinba-Ségard H, Jamet A, Euphrasie D, Durel B, Barois N, Pelissier P, Morand PC, Coureuil M, Lafont F, Join-Lambert O, Nassif X, Bourdoulous S. 2019. Targeting type IV pili as an antivirulence strategy against invasive meningococcal disease. Nat Microbiol 4:972–984. doi:10.1038/s41564-019-0395-8 30911127

[20] Vaziri H, Benchimol S. 1998. Reconstitution of telomerase activity in normal human cells leads to elongation of telomeres and extended replicative life span. Curr Biol 8:279–282. doi:10.1016/s0960-9822(98)70109-5 9501072

[21] Knetzger N, Regensburger AK, Goy A, Han H, Theuersbacher J, Tarau IS, Cleve C, Königer L, Finger T, Eibichova S, Haider MS, Schwarz T, Hansmann J, Hillenkamp J, Kampik D, Lotz C. 2025. From injury to recovery: investigating wound healing in a 3D tissue-engineered cornea equivalent. Biomaterials 323:123409. doi:10.1016/j.biomaterials.2025.123409 40449080

[22] Jonsson AB, Ilver D, Falk P, Pepose J, Normark S. 1994. Sequence changes in the pilus subunit lead to tropism variation of Neisseria gonorrhoeae to human tissue. Mol Microbiol 13:403–416. doi:10.1111/j.1365-2958.1994.tb00435.x 7997158

[23] Zöllner R, Cronenberg T, Kouzel N, Welker A, Koomey M, Maier B. 2019. Type IV pilin post-translational modifications modulate material properties of bacterial colonies. Biophys J 116:938–947. doi:10.1016/j.bpj.2019.01.020 30739725 PMC6400827

[24] Sadarangani M, Pollard AJ, Gray-Owen SD. 2011. Opa proteins and CEACAMs: pathways of immune engagement for pathogenic Neisseria. FEMS Microbiol Rev 35:498–514. doi:10.1111/j.1574-6976.2010.00260.x 21204865

[25] Edwards VL, Wang L-C, Dawson V, Stein DC, Song W. 2013. Neisseria gonorrhoeae breaches the apical junction of polarized epithelial cells for transmigration by activating EGFR. Cell Microbiol 15:1042–1057. doi:10.1111/cmi.12099 23279089 PMC5584544

[26] Rodríguez-Tirado C, Maisey K, Rodríguez FE, Reyes-Cerpa S, Reyes-López FE, Imarai M. 2012. Neisseria gonorrhoeae induced disruption of cell junction complexes in epithelial cells of the human genital tract. Microbes Infect 14:290–300. doi:10.1016/j.micinf.2011.11.002 22146107

[27] Wang L-C, Yu Q, Edwards V, Lin B, Qiu J, Turner JR, Stein DC, Song W. 2017. Neisseria gonorrhoeae infects the human endocervix by activating non-muscle myosin II-mediated epithelial exfoliation. PLoS Pathog 13:e1006269. doi:10.1371/journal.ppat.1006269 28406994 PMC5391109

[28] Das S, D’Souza S, Gorimanipalli B, Shetty R, Ghosh A, Deshpande V. 2022. Ocular surface infection mediated molecular stress responses: a review. Int J Mol Sci 23:3111. doi:10.3390/ijms23063111 35328532 PMC8952005

[29] Jongkhajornpong P, Nakamura T, Sotozono C, Inatomi T, Kinoshita S. 2015. Phenotypic investigation of regenerated epithelial cells after gonococcal corneal perforation: a clinical, histological, and immunohistochemical study. Cornea 34:1508–1512. doi:10.1097/ICO.0000000000000551 26203757

[30] Plant LJ, Jonsson A-B. 2006. Type IV pili of Neisseria gonorrhoeae influence the activation of human CD4^+^ T cells. Infect Immun 74:442–448. doi:10.1128/IAI.74.1.442-448.2006 16369000 PMC1346638

[31] Makepeace BL, Watt PJ, Heckels JE, Christodoulides M. 2001. Interactions of Neisseria gonorrhoeae with mature human macrophage opacity proteins influence production of proinflammatory cytokines. Infect Immun 69:1909–1913. doi:10.1128/IAI.69.3.1909-1913.2001 11179372 PMC98101

[32] Ramsey KH, Schneider H, Cross AS, Boslego JW, Hoover DL, Staley TL, Kuschner RA, Deal CD. 1995. Inflammatory cytokines produced in response to experimental human gonorrhea. J Infect Dis 172:186–191. doi:10.1093/infdis/172.1.186 7797909

[33] Maisey K, Nardocci G, Imarai M, Cardenas H, Rios M, Croxatto HB, Heckels JE, Christodoulides M, Velasquez LA. 2003. Expression of proinflammatory cytokines and receptors by human fallopian tubes in organ culture following challenge with Neisseria gonorrhoeae. Infect Immun 71:527–532. doi:10.1128/IAI.71.1.527-532.2003 12496205 PMC143407

[34] Juárez O, Barquera B. 2012. Insights into the mechanism of electron transfer and sodium translocation of the Na^+^-pumping NADH:quinone oxidoreductase. Biochim Biophys Acta 1817:1823–1832. doi:10.1016/j.bbabio.2012.03.017 22465856 PMC8172247

